# Achieving Gold Standards in Ethics and Human Rights in Medical Practice

**DOI:** 10.1371/journal.pmed.0020260

**Published:** 2005-07-19

**Authors:** Solomon R Benatar

## Abstract

A new study suggests that some physicians in Nigeria fall short in their ethical conduct. Benatar urges caution in interpreting the study, saying that "comparisons should be made with other countries."

Chen Reis and colleagues' study in this month's *PLoS Medicine* showed that most Nigerian physicians commendably appeared to be providing appropriate care for HIV/AIDS patients [1]. However, 9% refused to care for such patients, 9% admitted they had refused a patient with HIV/AIDS admission to hospital, and 20% felt that many of these patients had behaved immorally and deserved the disease. The authors also noted the adverse impact of limited health care resources upon ethical practice and protection of human rights (this impact is not surprising in a very poor country with a per-capita GDP of US$290 [2], less than 1% of the United States per-capita GDP). They conclude that discriminatory behaviour and breaches of ethical codes could be addressed effectively through education, enforcement of anti-discrimination policies, increasing resources for health care, and attempts to change attitudes and cultural beliefs.

Presumably, their motivations for this study were (1) to better understand how well physicians in Nigeria respect human rights and meet universal ethical standards of medical practice in caring for patients with HIV/AIDS, and (2) to make recommendations that could improve professional practice. Their findings would be more convincing if they could compare their data with similar studies done elsewhere in the world, including the US (home to some of the authors of the study). In particular, it would be valuable to have comparative international data on ethics and human rights standards achieved in medical practice, and on health professionals' attitudes to patients with HIV/AIDS and other stigmatised conditions. However, Reis and colleagues' study raises several important questions.

## What Are the Gold Standards?

What are the gold standards against which to evaluate the ethics and human rights standards that physicians are expected to meet in practice? Presumably these are the standards set out in the declarations, codes, and guidelines quoted by Reis et al., and presumably nothing less than 100% compliance is acceptable. These standards are then applicable to all physicians everywhere—especially to those from rich countries, where the resources available for medical care and continuing education outstrip by orders of magnitude those available in very low income countries like Nigeria.

In order to fully understand the significance of the failure of some Nigerian physicians to meet these gold standards, substantive comparisons should be made with other countries. To do so would entail systematic studies of the extent to which physicians from the US and other wealthy countries meet the requirements of international and local codes of ethics and human rights. Military physicians in the US fall short of gold standards [3,4], and a third of US scientists have engaged in serious research misconduct in the past three years [5]. There is clearly a need for comparative studies in everyday practice.

## Do We Know What Causes Discriminatory Practices?

Reis and colleagues' study raises several questions about the basis for discriminatory practices—questions that the study itself cannot fully answer. In particular, how can we judge whether ethical shortcomings in physicians' behaviour (in Nigeria and elsewhere) are due to lack of appropriate health care facilities, inadequate education, lack of enforcement of anti-discriminatory policies, or cultural and other socially determined discriminatory attitudes that might persist despite adequate education and health care facilities?

In order to be able to explore these questions about causality, the authors would need to look at whether there is any evidence that standards of ethics and human rights achieved in practice correlate significantly with health care facilities, education about ethics and human rights, mechanisms for enforcing anti-discrimination policies, and cultural attitudes. Is there any comparative data, for example, on whether higher ethical and human rights standards are achieved in medical practice in wealthy industrialised countries as compared with poor countries, or in countries with universal access to health care as compared with privatised medicine?

It would also be valuable to consider whether health professionals' cultural attitudes about illness elsewhere in the world (not only in Nigeria) may contribute to stigma, discrimination, and worse patient care. Such attitudes could include the belief that medical care is a commodity that should be most accessible to those who can pay, that HIV/AIDS is punishment from God, and that patients who smoke, are obese, or have an unwanted pregnancy are all morally blameworthy and deserve their conditions. Surely such cultural attitudes, wherever they exist, are also worthy of study?

## How Can We Reduce Discriminatory Practices?

Reis and colleagues argue that there need to be more resources for HIV care in Nigeria. Their plea should be considered within the broader context of the perverse cultural attitudes that drive the global political economy, promote continuing extraction of human and material resources from developing countries, and sustain poverty [6,7]. Such cultural attitudes and practices that undermine health globally may in fact have a more widely corrosive effect on human rights and professional practices than anything Nigerians do.

If discriminatory practices could be reduced in Nigeria by attention to the rights of people living with AIDS, this implies that such attention could also improve the rights of patients anywhere. For example, in middle-income and wealthy countries, such attention might reduce stigmatisation and moral blaming, improve the rights of many who suffer from chronic “lifestyle diseases” (such as chronic lung diseases related to smoking), and prevent discrimination in access to health care for those who lack insurance cover. It would be valuable to compare Reis and colleagues' study with any studies done in middle-income and wealthy countries that examine whether attention to patient rights improves discriminatory practices.

## Conclusions

It is unlikely that any group of physicians anywhere in the world fully meets all of the ethical and human rights standards set in international guidelines. Studies of physicians' shortcomings should be universal. What should be avoided is the previous colonial mentality of wanting to study and improve others [8] while oblivious of the need to address the more sophisticated and covert faults of Western researchers' own societies. The desire to improve the behaviour of others should also be associated with awareness that one's own exemplary moral behaviour might be more effective in promoting ethical behaviour and respect for human rights [9,10] than exhortation, “education” and attempts to change the cultural attitudes of others while neglecting our own adverse cultural attitudes.
